# Physicochemical and Sensory Evaluation of Romanian Monofloral Honeys from Different Supply Chains

**DOI:** 10.3390/foods14132372

**Published:** 2025-07-04

**Authors:** Elena Daniela Bratosin, Delia Mirela Tit, Manuela Bianca Pasca, Anamaria Lavinia Purza, Gabriela Bungau, Ruxandra Cristina Marin, Andrei Flavius Radu, Daniela Gitea

**Affiliations:** 1 Doctoral School of Biological and Biomedical Sciences, University of Oradea, 410087 Oradea, Romania; bratosin.elenadaniela@student.uoradea.ro (E.D.B.); gbungau@uoradea.ro (G.B.); marin.ruxandracristina@student.uoradea.ro (R.C.M.); andreiflavius.radu@uoradea.ro (A.F.R.); 2 Department of Pharmacy, Faculty of Medicine and Pharmacy, University of Oradea, 410028 Oradea, Romania; purza.anamarialavinia@didactic.uoradea.ro (A.L.P.); dgitea@uoradea.ro (D.G.); 3 Department of Psycho-Neurosciences and Recovery, Faculty of Medicine and Pharmacy, University of Oradea, 410073 Oradea, Romania

**Keywords:** honey, floral origin, palynological analysis, physicochemical parameters, sugar profile, sensory evaluation

## Abstract

Honey quality and authenticity are influenced by floral origin, processing, and storage, with implications for composition and sensory appeal. This study offers a comparative assessment of eight monofloral honey samples, representing five botanical varieties: acacia, linden, rapeseed, lavender, and thyme. For acacia, linden, and rapeseed, both producer-sourced and commercial honeys were analyzed, while lavender and thyme samples were available only from local beekeepers. The botanical origin of each sample was confirmed using morphological markers of pollen grains. Physicochemical characterization included acidity, pH, moisture content, refractive index, hydroxymethyl furfural (HMF), proline concentration, and carbohydrate profiling by HPLC-RID. Acacia honey exhibited the lowest acidity and HMF levels, alongside the highest fructose/glucose (F/G) ratios, indicating superior freshness, lower crystallization risk, and a sweeter flavor profile. In contrast, rapeseed honey showed elevated glucose levels and the lowest F/G ratio, confirming its tendency to crystallize rapidly. All samples recorded proline concentrations well above the quality threshold (180 mg/kg), supporting their authenticity and proper maturation. The estimated glycemic index (eGI) varied between 43.91 and 62.68 and was strongly inversely correlated with the F/G ratio (r = −0.98, *p* < 0.001). Sensory evaluation highlighted acacia honey from producers as the most appreciated across visual, tactile, and flavor attributes. Correlation analyses further revealed consistent links between sugar composition and both physical and sensory properties. Overall, the findings reinforce the value of integrated analytical and sensory profiling in assessing honey quality and authenticity.

## 1. Introduction

Honey has been appreciated for more than two millennia in the territory of Romania, with the ancient Dacians being recognized for producing high-quality honey—a natural product renowned for its nutritional and therapeutic properties which continues to hold significant value in worldwide agriculture and human health [1,2]. In 2023, the global honey market achieved a revenue of approximately USD 9.3 billion, reflecting a growing consumer preference for natural and organic sweeteners. This surge has been driven by honey’s multifaceted health benefits, including its antioxidant, antimicrobial, and anti-inflammatory properties, which contribute to its role in promoting cardiovascular health, supporting immune function, and aiding in wound healing [3,4].

According to European Directive 2001/110/EC, honey is defined as the natural sweet substance produced by *Apis mellifera* bees from nectar or plant secretions, which is subsequently processed, stored, and matured in honeycombs [5]. The Codex Alimentarius offers a similar definition, with broader reference to “honey bees,” recognizing global diversity in bee species involved in honey production [6,7].

Romania holds a prominent position among European honey producers, contributing significantly to the apiculture sector. However, in 2024, honey production in Romania experienced a dramatic decline of approximately 40–45%, driven by extreme weather conditions that severely impacted floral sources such as rapeseed, acacia, linden, and sunflower [8]. This environmental vulnerability raises pressing concerns about the sustainability of apiculture under accelerating climate change.

Beyond its nutritive and economic roles, honey has a long-standing place in traditional medicine, where it has been used to treat a wide range of ailments, from respiratory and gastrointestinal disorders to skin diseases and infections [9]. Scientific studies confirm that honey possesses a complex biochemical profile comprising organic acids, polyphenols, amino acids, enzymes, vitamins, and minerals, all of which contribute to its therapeutic potential [10,11,12,13,14]. Different floral origins can confer distinct physicochemical and sensory characteristics to honey, including variations in acidity, sugar content, mineral composition, and antioxidant activity [15,16].

Honey is classified as either monofloral, derived predominantly from a single plant species, or multifloral, depending on the diversity of floral sources accessed by bees [17]. Quality standards established at international and national levels assess key parameters such as moisture content, electrical conductivity, sugar composition (notably fructose and glucose), enzymatic activity (e.g., diastase, invertase), proline levels, and hydroxymethyl furfural (HMF). Moreover, health-related safety assessments often include screening for heavy metals (e.g., lead and cadmium), pesticide residues, and microbial contamination [5,17]. The physicochemical attributes of honey are highly sensitive to variables such as botanical source, environmental conditions, bee species, harvesting period, processing techniques, and storage duration. Consequently, parameters like pH, moisture, electrical conductivity, and mineral profile can vary widely, even among samples of the same floral origin [18,19]. Several studies have highlighted that honey’s composition is not only influenced by its floral source but also by geographic origin, with clear implications for consumer preferences and market value [20,21]. In particular, physicochemical traits (sugar profile, proline concentration, acidity, and HMF content) play a key role in both sensory perception and nutritional classification. Understanding how these traits vary according to botanical origin is essential for ensuring product quality, guiding consumer choices, and protecting the integrity of the honey supply chain.

In light of these considerations, this study aims to investigate how floral origin influences the physicochemical composition and sensory characteristics of Romanian monofloral honey. Building on recent advances in honey profiling and traceability, the research also examines whether samples collected from local beekeepers and commercial retailers exhibit comparable patterns in quality parameters and consumer-perceived attributes.

## 2. Materials and Methods

### 2.1. Sample Collection and Classification

In this study, five types of monofloral honey were analyzed, acacia (*Robinia pseudoacacia* L.), rapeseed (*Brassica napus* L.), linden (*Tilia* spp.), lavender (*Lavandula angustifolia* L.), and thyme (*Thymus* spp.). Samples were sourced from two main channels. First, raw and unprocessed honey was obtained directly from local beekeepers participating in a national fair dedicated to certified producers. These were referred to as producer samples (marked “_P”) and were harvested in the same year they were acquired (2022). Second, commercial samples (marked “_C”) were purchased from retail outlets, representing honey already processed and marketed to the public. It is important to note that for lavender (LH_P) and thyme (TH_P) honey, only producer samples were included in this study. Despite extensive efforts, no commercial samples of these two floral types were found on the Romanian retail market at the time of sampling. As such, their inclusion was limited to raw, unprocessed honeys sourced directly from certified local beekeepers. This selection reflects the current limited commercial availability of lavender and thyme monofloral honeys, which, although highly valued, are produced in small quantities and rarely reach mainstream distribution channels.

The producer samples were sourced from different regions of Romania, specifically from counties located in the south-east (Brăila, Buzău), north-east (Vaslui), and north-west (Sălaj), based on the availability of specific floral types at the time of collection. These locations are reported for transparency but were not selected to systematically represent specific geographical regions. All samples were stored in sealed glass containers under consistent laboratory conditions (dark environment, 20 ± 2 °C, and relative humidity below 50%) and were analyzed without further processing. The classification of honey by floral type and source (producer vs. commercial) is shown in Table 1.

### 2.2. Reagents and Materials

Sodium hydroxide and phenolphthalein, both of analytical grade, were obtained from Sigma-Aldrich (Darmstadt, Germany). Carrez I reagent (15 g/100 mL of potassium hexacyanoferrate (II) trihydrate) and Carrez II reagent (30 g/100 mL of zinc acetate dihydrate) were freshly prepared using reagents from Sigma-Aldrich (Darmstadt, Germany). Sodium bisulfite (NaHSO_3_), analytical grade, was also supplied by Sigma-Aldrich (Germany). Ninhydrin (≥99%) was sourced from Carl Roth GmbH + Co. KG (Karlsruhe, Germany) and dissolved in ethylene glycol. Formic acid p.a. (80% solution) and fuchsin were acquired from Merck (Darmstadt, Germany), and isopropanol (50% *v*/*v*, analytical grade) was used as a diluent. Hydrochloric acid (HCl) and sulfuric acid (H_2_SO_4_) were obtained from Chempur (Piekary Śląskie, Poland). Ultrapure water was produced using a Direct-Q UV purification system (Millipore Corporation, Billerica, MA, USA). Analytical-grade carbohydrate standards, including maltose, sucrose, glucose, and fructose (≥99% purity), were purchased from Merck (Darmstadt, Germany).

### 2.3. Identification of Floral Origin of Honey Samples

The floral origin of each honey sample was confirmed by the microscopic examination of pollen grains. For sample preparation, approximately 10 g of honey was weighed and dissolved in distilled water at room temperature to a final volume of 30 mL. Samples were centrifuged twice with a Hettich EBA 200 centrifuge (Andreas Hettich GmbH & Co. KG, Tuttlingen, Germany) at 4500 rpm and 900× *g* for 10 min (first centrifugation), and then for 5 min (second centrifugation) at the same force. The sediment was examined microscopically under 10×, 40×, and 100× objectives. Glass slides, slides, and aqueous fuchsin solution were used to stain the samples. For determining the floral sources of the 8 samples of honey under study, the morphological characteristics of the pollen grains were analyzed by optical microscopy C-B10+ (Optika S.r.l., Ponteranica, Italy; ZIP 24010), equipped with an Optica B10 digital camera was used to make observations and capture pictures.

Pollen types were identified by comparing their shape, symmetry, aperture type, and exine ornamentation with reference atlases for Romanian melliferous flora. Based on these characteristics, all samples were classified as monofloral, corresponding to one of five plant species (*Robinia pseudoacacia* L., *Brassica napus* L., *Tilia* spp., *Lavandula angustifolia* L., or *Thymus* spp.). In several cases, particularly for acacia and rapeseed honeys, only pollen from the declared floral source was observed, with no detectable presence of secondary pollen types. In other samples, small amounts of secondary pollen (e.g., from sunflower, chicory, or Salvia species) were detected, likely due to overlapping flowering periods, but these did not compromise the dominant floral identity.

### 2.4. Determination of pH and Titratable Acidity in Honey Samples

Approximately 10 g of each honey sample was dissolved in 75 mL of distilled water, and the pH of the resulting solution was measured using a calibrated pH meter. Titratable acidity (*TA*) was then determined by titration with 0.1 M sodium hydroxide (NaOH) in the presence of phenolphthalein as an indicator until a persistent pale pink coloration was observed. The titratable acidity was calculated (1) using Equation (1), and the results were expressed in milliequivalents of acid per kilogram of honey:

(1) $$TA=\frac{V\times 0.1\times 0.064\times 1000}{g}$$

where *V* = the volume of 0.1 M NaOH used for titration (mL); 0.1 = the molarity of the NaOH solution; 0.064 = the specific conversion factor from NaOH volume to acid equivalents; 1000 = the factor used to standardize results to 100 g of product; and *g* = the grams of honey used in the determination [22,23].

### 2.5. Determination of Moisture Content and Total Solids

The moisture content of the honey samples was determined by measuring the refractive index at 20 °C using an Abbe refractometer (model 2WAJ, OPTIKA, Ponteranica, Italy). The moisture percentage (g/100 g of honey) was then calculated using standard reference tables published by the International Honey Commission (IHC). The total solids (TS) content was determined by subtracting the moisture content from 100%, according to Equation (2) [24]:

(2) *TS* (%) = 100 – *Moisture* (%)

### 2.6. Determination of Hydroxymethyl Furfural (HMF) Content

The content of HMF in the honey samples was determined according to AOAC Official Method 980.23 (1983). Briefly, 5 g of honey was dissolved in 25 mL of distilled water. Subsequently, 0.5 mL each of Carrez I and Carrez II reagents were added, and the mixture was brought to volume with distilled water in a 50 mL volumetric flask. The solution was filtered through filter paper, and the first 10 mL of the filtrate was discarded. Two aliquots of 5 mL each were transferred into separate test tubes. In the first tube (sample solution), 5 mL of distilled water was added. In the second tube (reference solution), 5 mL of 0.20% sodium bisulfite solution was added. The absorbance of both solutions was measured at 284 nm and 336 nm using an ultraviolet–visible (UV-vis) spectrophotometer (PG Instruments Ltd., Leicestershire, UK). The HMF content was calculated by applying Equation (3), with the results expressed in mg/kg:

(3) *HMF* (mg/kg) *=* (*A*_284_) − (*A*_336_) *×* 149.7

where *A*_284_ = the absorbance at 284 nm; *A*_336_ = the absorbance at 336 nm; and 149.7 = a coefficient based on sample mass and the molecular weight of HMF [25].

### 2.7. Determination of Carbohydrates by HPLC-RID

#### 2.7.1. Sample Preparation and Carbohydrate Extraction

Carbohydrate extraction was carried out using acidified water (pH 2, adjusted with 1M hydrochloric acid (HCl)). Precisely 1 g of honey was weighed into a 15 mL centrifuge tube, and 5 mL of pH 2 water was added. The mixture was vortexed for 1 min using a Heidolph Reax Top vortex mixer, followed by sonication for 15 min in an Elmasonic E 15 H ultrasonic bath. The sample was then centrifuged at 1700× *g* for 10 min at 24 °C using an Eppendorf AG 5804 centrifuge. The resulting supernatant was filtered through a Chromafil Xtra nylon membrane filter (0.45 µm pore size), and a 20 µL aliquot was injected into an HPLC system for carbohydrate analysis [26].

#### 2.7.2. Chromatographic Conditions for Carbohydrate Analysis

Carbohydrate separation and quantification were performed using an Agilent 1200 HPLC system (Agilent Technologies, Santa Clara, CA, USA) equipped with a quaternary pump, solvent degasser, manual injector, and a refractive index detector (RID). The separation was carried out on a Polaris Hi-Plex H column (300 × 7.7 mm, Agilent Technologies, Santa Clara, CA, USA) using 5 mM sulfuric acid (H_2_SO_4_) as the mobile phase at a flow rate of 0.6 mL/min. The column was maintained at 70 °C, and the RID was set at 35 °C. The total run time for each analysis was 25 min. Data acquisition and chromatographic analysis were conducted using OpenLab—ChemStation software (version C.01.09, Agilent Technologies, Santa Clara, CA, USA) [27]. The identification of carbohydrates in the honey samples was based on the comparison of retention times with those of authentic standard compounds.

### 2.8. Estimation of Glycemic Index

Theoretical glycemic index (GI) was calculated using the sugar content established by HPLC. The computation employed the individual concentrations of the primary monosaccharides and disaccharides that contribute to honey’s postprandial glycemic impact. The following sugars were assigned a reference glycemic index value from the literature: glucose (GI = 100), fructose (GI = 19), sucrose (GI = 65), and maltose (GI = 105) [28,29]. The estimated GI (eGI) for each sample was calculated using a weighted average method that considered the contribution of each sugar to total carbohydrate content:

(4) *eGI* = [(*G* × 100) + (*F* × 19) + (*S* × 65) + (*M* × 105)]/*TC*

where *G* = glucose content, *F* = fructose, *S* = sucrose, *M* = maltose, and *TC* = total carbohydrate content in the sample. This technique, however theoretical, has been verified in prior research as a good proxy for glycemic potential when in vivo glycemic index testing is not practical due to logistical or ethical restrictions [30].

The fructose-to-glucose ratio (*F/G*) was determined as an additional predictor of glycemic behavior, given that a higher fructose content (in relation to glucose) is often linked with a lower glycemic response due to its slower absorption and hepatic processing [31,32].

### 2.9. Determination of Proline Content

The proline concentration was determined using a colorimetric method based on the reaction with ninhydrin and calculated using Equation (5):

(5) *Proline* (mg/kg) = (*h_a_*/*s_a_*) × (*s_p_*_1_/*s_p_*_2_) × 80

where h_a_ = the absorbance of the honey sample; s_a_ = the absorbance of the standard proline solution; s_p1_ = the amount of proline (mg) in the standard solution; s_p2_ = the amount of honey (g) used in the analysis; and 80 = an empirical constant that converts absorbance data into proline concentration expressed in mg/kg of honey.

For analysis, 0.5 mL of honey solution, 1 mL of 3% ninhydrin in ethylene glycol, and 1 mL of 80% formic acid were added to a test tube. The mixture was stirred and then heated in a boiling water bath for 10 min. Afterward, 5 mL of 50% isopropanol was added, and the solution was allowed to react. The absorbance was measured at 510 nm using ultraviolet–visible (UV-vis) spectrophotometer (PG Instruments Ltd., Leicestershire, UK). Quantification was performed by comparing the absorbance of the sample against that of a standard proline solution [33].

### 2.10. Sensory Evaluation

The panel consisted of 30 young adult consumers (aged 20–24), recruited from university students based on their availability and willingness to participate. The evaluated attributes included color, crystallization, aroma, texture, and overall flavor. The assessment was structured as a hedonic test, focusing on two main categories of attributes: visual–tactile aspects (such as color, crystallization, texture, and ease of spreading) and gustatory–olfactory characteristics (specifically aroma and flavor perceived during tasting). Each attribute was rated using a standardized 9-point hedonic scale, where a score of 9 represented “extremely pleasant,” 5 indicated a neutral response, and 1 corresponded to “extremely unpleasant.” This scale allowed participants to express their level of acceptance for each honey sample in a consistent and quantifiable manner [34,35]. Although participants were not trained sensory professionals, they received a short familiarization session before the test, in which they were introduced to the evaluated attributes and instructed on how to use the 9-point hedonic scale. Each sample was evaluated monadically in standardized glass vials (40 mL) labeled with random alphanumeric codes and served at room temperature. The sensory sessions were conducted in a dedicated space, separated from the preparation area. Panelists rinsed their mouths with water between samples.

To minimize sensory carry-over and positional bias, samples were grouped by floral type and balanced within each group to alternate source order (producer vs. commercial). For example, participants evaluated the acacia honey samples (AH_P and AH_C) one after the other, followed by the linden and rapeseed pairs, lavender, and thyme. This approach was chosen to support the more focused perception of subtle differences within the same botanical group while avoiding the sensory confusion that might arise from switching between distinct floral types.

The focus of the test was to capture spontaneous consumer-level impressions of honey characteristics and acceptability, rather than to build a descriptive sensory profile. As such, no calibration or consensus training was performed, and the results were interpreted in the context of consumer acceptability, not expert sensory profiling.

The Research Ethics Committee of the Faculty of Medicine and Pharmacy, University of Oradea, Oradea, Romania, assessed and approved the sensory analysis procedure (No. CEFMF/3 of 22 December 2022). All participants provided written informed permission, and the processes adhered to the established protocol.

### 2.11. Statistical and Correlation Analysis

All statistical analyses were performed using JASP software (version 0.19.3.0), following the centralization of experimental data in Microsoft Excel. To evaluate differences among honey types and between source categories (producer vs. commercial), one-way analysis of variance (ANOVA) was applied, followed by Tukey’s Honest Significant Difference (HSD) post hoc test for pairwise comparisons. Results were expressed as means ± standard deviation, and statistical significance was considered at a threshold of *p* < 0.05. Differences among means were denoted by distinct superscript letters. In addition, Pearson correlation analysis was conducted to assess linear relationships between physicochemical parameters. Correlation strength was interpreted based on the value of the coefficient (r), with the thresholds set as follows: moderate (|r| = 0.40–0.59), strong (|r| = 0.60–0.79), and very strong (|r| ≥ 0.80). Statistical significance was evaluated at *p* < 0.05, *p* < 0.01, and *p* < 0.001. For relationships between sensory attributes and analytical parameters, Kendall’s Tau correlation was employed, suitable for ordinal sensory data. All figures were generated within JASP.

## 3. Results

### 3.1. Microscopic Characterization of Pollen Morphology

**Figure 1:**
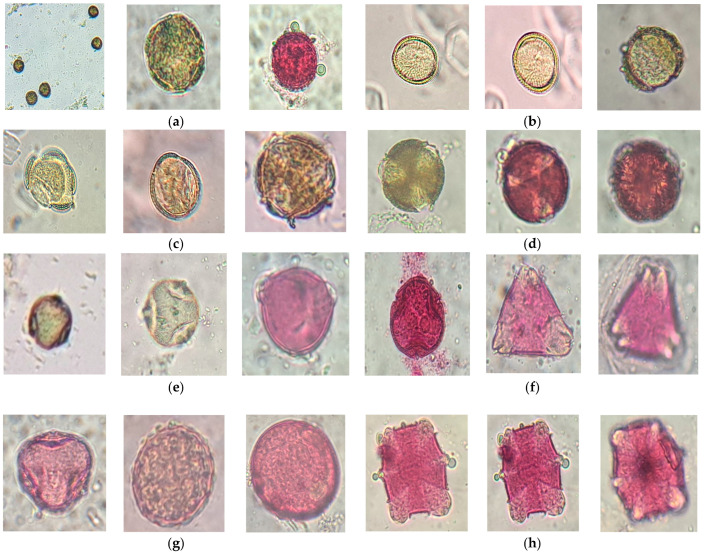
Microscopic images of pollen sediment from monofloral honey samples: (**a**) AH_P, producer acacia honey; (**b**) AH_C, commercial acacia honey; (**c**) RH_P, producer rapeseed honey; (**d**) RH_C, commercial rapeseed honey; (**e**) TLH_P, producer linden honey; (**f**)—TLH_P, commercial linden honey; (**g**) LH_P, producer lavender honey; (**h**) TH_P, producer thyme honey.

Acacia honey (AH_P and AH_C) contained exclusively pollen from *Robinia pseudoacacia* L. These grains exhibited spheroidal to slightly oval shapes, isopolar symmetry, and a smooth or slightly granular exine surface. The aperture was triporate, with three small circular pores that facilitate germination, although they were sometimes difficult to observe clearly. The unstained pollen appeared light yellow to golden-brown and darkened noticeably upon fuchsin staining. Both samples showed identical morphological characteristics, and no other pollen types were observed (Figure 1a,b).

Rapeseed honey (RH_P and RH_C) was dominated by pollen from *Brassica napus* L. The grains were nearly spherical, with a slightly elongated subprolate form and isopolar symmetry. Their exine displayed a delicate reticulate pattern resembling a fine mesh. A tricolporate aperture, three elongated furrows with central pores, was clearly visible. These grains lacked spines and shifted from light yellow to maroon when stained (Figure 1c,d). No secondary pollen was present in either sample.

Linden honey (TLH_P and TLH_C) primarily contained pollen from Romanian *Tilia* species, including *T. cordata, T. tomentosa,* and *T. platyphyllos*. The grains were round to slightly oval, with symmetrical apical and lateral views, and featured a smooth, finely reticulated exine. A tricolporate aperture was consistently observed. The pollen had a bright yellow-green hue, turning cyclamen pink when treated with fuchsin (Figure 1e,f). A small number of grains with spiny tricolporate ornamentation, likely from *Helianthus annuus* L. (sunflower) or *Cichorium intybus* L. (chicory), which bloom concurrently with linden, were also present.

Lavender honey (LH_P) was characterized by pollen from *Lavandula angustifolia* L., presenting predominantly spheroidal to slightly oval grains with isopolar symmetry. The exine featured a fine reticulated or perforated texture, and the tricolporate aperture supported accurate identification. These grains were pale yellow in their natural state and turned rosy upon fuchsin staining (Figure 1g). Additionally, a limited amount of tricolporate pollen with spiny ornamentation, likely from sunflower or chicory, was identified, consistent with the flowering period overlap.

Thyme honey (TH_P) contained pollen specific to *Thymus* spp. The grains were slightly elliptical (prolatoid), with isopolar symmetry and a granuloreticulate to microreticulate exine pattern. A hexacolporate aperture morphology, consisting of six elongated furrows with central pores, was observed and consistently identified across multiple grains. These grains lacked spines and shifted from pale yellow to pink when stained (Figure 1h). Two additional types of pollen were present: spiny tricolporate grains, likely from sunflower or chicory, and fine tricolporate grains that may have originated from Salvia species.

### 3.2. Acidity and pH of Honey

Acacia honey exhibited the lowest acidity values, both in the producer sample (7.73 mEq/kg) and the commercial counterpart (7.84 mEq/kg). The corresponding pH values were also low, ranging from 3.83 to 3.85, indicating a slightly acidic medium, as expected in fresh, unfermented honey. On the other hand, the lavender (21.40 mEq/kg) and thyme honeys (21.39 mEq/kg) showed the highest acidity values, suggesting a more active biochemical composition, possibly influenced by their rich content of organic acids, volatile aromatic compounds, or the specific morphology of pollen grains associated with these floral sources. Similarly, rapeseed honey demonstrated elevated acidity (ranging from 19.73 to 21.21 mEq/kg), further supporting the connection between floral source and acid content. Linden honey, regardless of origin, presented intermediate acidity values (15.71–17.43 mEq/kg), but distinguished itself by recording the highest pH, reaching up to 5.06 in the commercial sample (Table 2). This relatively higher pH may be attributed to the presence of buffering compounds such as minerals and polyphenols, which are commonly associated with this floral variety. Overall, the acidity and pH profiles of the honey samples from commercial sources were comparable to those of the raw samples obtained directly from producers. All the measured titratable acidity values fell well within the regulatory limit of 50 milliequivalents/kg established by EU legislation and Codex Alimentarius.

**Table 2:** Acidity and pH values of honey samples.

Honey Sample	Acidity mEq/kg	pH
AH_P	7.73 ± 0.03 ^a^	3.83 ± 0.05 ^ab^
AH_C	7.84 ± 0.06 ^a^	3.85 ± 0.06 ^ab^
RH_P	21.21 ± 0.02 ^e^	3.72 ± 0.08 ^a^
RH_C	19.73 ± 0.12 ^d^	4.05 ± 0.06 ^bc^
TLH_P	17.43 ± 0.11 ^c^	5.06 ± 0.14 ^d^
TLH_C	15.71 ± 0.10 ^b^	4.20 ± 0.10 ^c^
LH_P	21.40 ± 0.11 ^e^	3.69 ± 0.10 ^a^
TH_P	21.39 ± 0.02 ^e^	3.81 ± 0.10 ^ab^

Results presented as means ± SD (*n* = 3). ^a–e^—different lowercase letters indicate significant differences between samples (*p* < 0.05) according to Turkey’s multiple comparison test. Samples sharing same letter are not significantly different from each other. AH_P, producer acacia honey; AH_C, commercial acacia honey; RH_P, producer rapeseed honey; RH_C, commercial rapeseed honey; TLH_P, producer linden honey; TLH_P, commercial linden honey; LH_P, producer lavender honey; TH_P, producer thyme honey.

### 3.3. Moisture and Total Solids

Thyme honey had the highest refractive index (1.5054), associated with the lowest water content (16.25 g/100 g), indicating a well-matured product with low fermentation risk.

Conversely, the rapeseed honey from producers recorded the lowest refractive index (1.4875) and the highest water content (19.60 g/100 g), suggesting incomplete extraction or maturation. However, its commercial counterpart exhibited a higher refractive index (1.4946), like the other varieties.

The linden and acacia honeys from both sources showed consistent water contents below 18%, indicating good product stability (Table 3). Generally, the producer samples showed slight parameter variability, possibly due to differences in extraction standardization and storage conditions. However, all measured water content values were below the regulatory maximum of 20 g/100 g, indicating proper dehydration and stability across all samples.

**Table 3:** Refractive index and water content of honey samples.

Honey Sample	Refractive Index n^D^ 20 °C	Water Content (g/100 g)
AH_P	1.4946 ^d^	16.80 ± 1.1 ^ab^
AH_C	1.4925 ^e^	17.60 ± 0.35 ^bc^
RH_P	1.4875 ^a^	19.60 ± 0.3 ^e^
RH_C	1.4946 ^e^	16.80 ± 0.6 ^ab^
TLH_P	1.4951 ^e^	16.60 ± 0.1 ^ab^
TLH_C	1.4910 ^c^	18.20 ± 0.55 ^cd^
LH_P	1.4885 ^b^	19.20 ± 0.1 ^de^
TH_P	1.5054 ^f^	16.25 ± 0.05 ^a^

Results presented as means ± SD (n = 3). ^a–f^—different lowercase letters indicate significant differences between samples (*p* < 0.05) according to Turkey’s multiple comparison test. Samples sharing same letter are not significantly different from each other. AH_P, producer acacia honey; AH_C, commercial acacia honey; RH_P, producer rapeseed honey; RH_C, commercial rapeseed honey; TLH_P, producer linden honey; TLH_P, commercial linden honey; LH_P, producer lavender honey; TH_P, producer thyme honey.

### 3.4. Hydroxymethyl Furfural (HMF) Content

**Figure 2:**
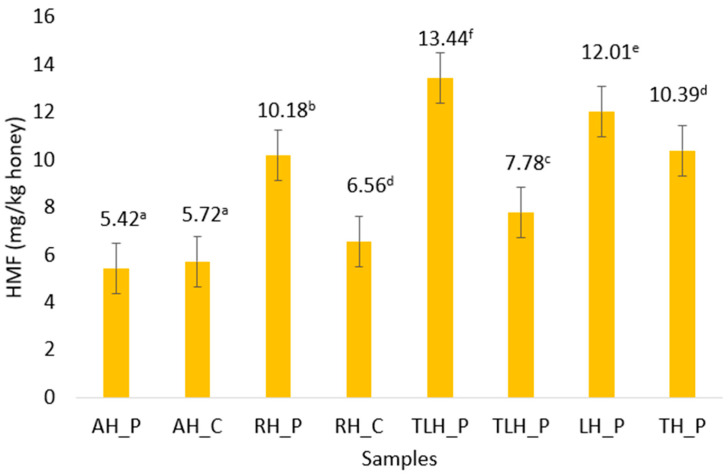
HMF content in honey samples. Results presented as means ± SD (n = 3). ^a–f^—different lowercase letters indicate significant differences between samples (*p* < 0.05) according to Turkey’s multiple comparison test. Samples sharing same letter are not significantly different from each other; AH_P, producer acacia honey; AH_C, commercial acacia honey; RH_P, producer rapeseed honey; RH_C, commercial rapeseed honey; TLH_P, producer linden honey; TLH_P, commercial linden honey; LH_P, producer lavender honey; TH_P, producer thyme honey.

The HMF content varied significantly among the analyzed honey samples, with values ranging from 5.42 to 13.44 mg/kg. The lowest levels were recorded in the acacia honey, both from the producer and the commercial source, with values below 7 mg/kg. The highest HMF content was observed in the linden honey from the producer at 13.44 mg/kg, significantly higher than its market counterpart. Elevated HMF levels were also recorded in the lavender and thyme honey, although no commercial counterparts were available for direct comparison. All samples remained well below the EU legal limit of 40 mg/kg, confirming compliance with quality standards. As shown in Figure 2, statistically significant differences (*p* < 0.05) were observed not only between producer and commercial variants of the same floral type, but also across all botanical origins.

### 3.5. Carbohydrate Composition, HPLC-RID Method

**Figure 3:**
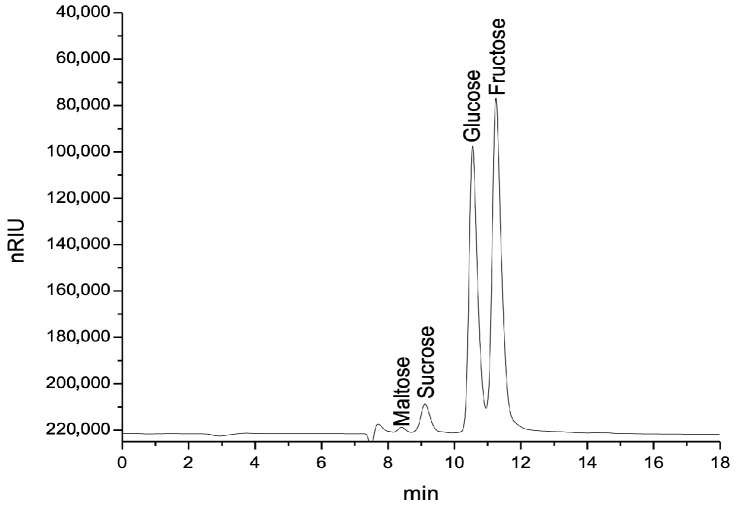
Chromatogram of producer acacia honey.

The sugar profiles of the honey samples revealed substantial differences both across floral types and between commercial and producer sources. Fructose and glucose were the predominant monosaccharides in all samples, while maltose and sucrose appeared in minor concentrations. Figure 3 illustrates a representative chromatogram obtained for acacia honey (producer source), highlighting the clear resolution of the main sugar peaks.

As shown in Table 4, all samples exceeded the minimum required threshold of 60 g/100 g for combined fructose and glucose content, as specified by EU and Codex Alimentarius regulations. The rapeseed honey, particularly from producers, recorded the highest combined sugar levels (80.10 g/100 g) but the lowest F/G ratio (0.89), confirming its strong tendency to crystallize quickly. In contrast, the acacia honeys displayed the highest F/G ratios (2.06 in producer and 2.44 in commercial sample), coupled with slightly lower combined fructose and glucose levels (approximately 70 g/100 g). Linden honey showed a moderate F/G ratio in both variants, but a noticeable source difference was observed. The producer sample had a higher F/G (1.86) than the commercial one (1.30), suggesting a more fluid and stable structure in the former. The lavender and thyme honeys, available only from producers, displayed intermediate F/G values (1.43 and 1.24) and a balanced sugar composition (fructose + glucose content between 74 and 75 g/100 g), suggesting moderate crystallization behavior and good stability.

**Figure 4:**
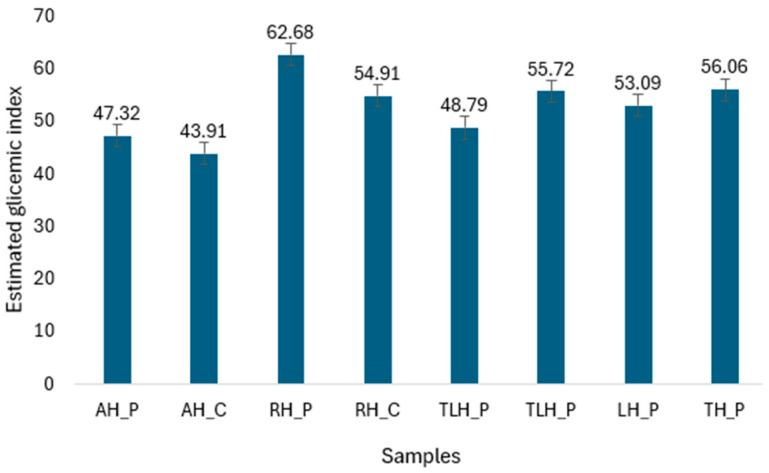
The estimated glycemic index (eGI) values. AH_P, producer acacia honey; AH_C, commercial acacia honey; RH_P, producer rapeseed honey; RH_C, commercial rapeseed honey; TLH_P, producer linden honey; TLH_P, commercial linden honey; LH_P, producer lavender honey; TH_P, producer thyme honey.

The estimated GI values ranged from 43.91 to 62.68, reflecting moderate to high potential glycemic responses depending on their sugar composition (Figure 4). The sample with the lowest eGI (43.91) corresponded to the highest fructose-to-glucose ratio (2.44), whereas the sample with the highest eGI (62.68) showed the lowest F/G ratio (0.89).

**Figure 5:**
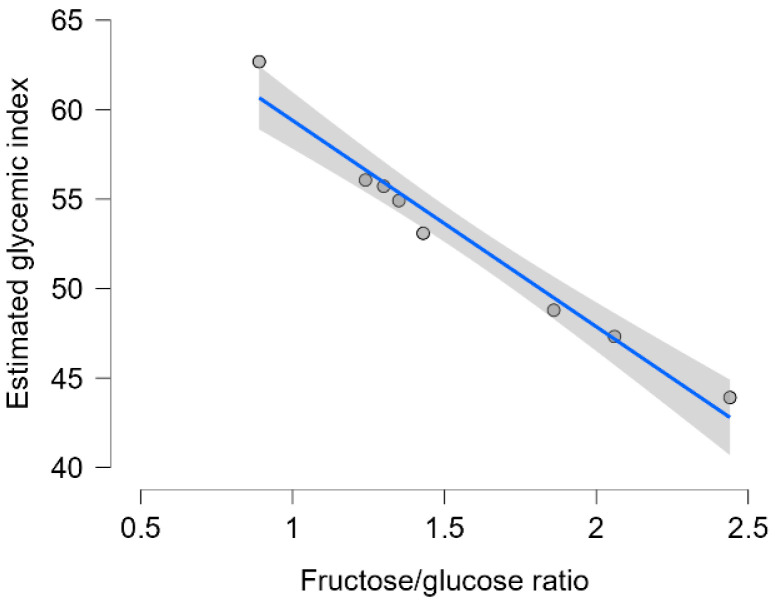
The correlation between the honey samples’ estimated glycemic index and fructose/glucose ratio.

Pearson correlation indicated a strong and statistically significant negative correlation between the two variables eGI and G/F ratio (r = −0.98, *p* < 0.001) and confirmed that honey samples with higher F/G ratios tended to exhibit lower estimated glycemic responses (Figure 5).

### 3.6. Proline Content

**Figure 6:**
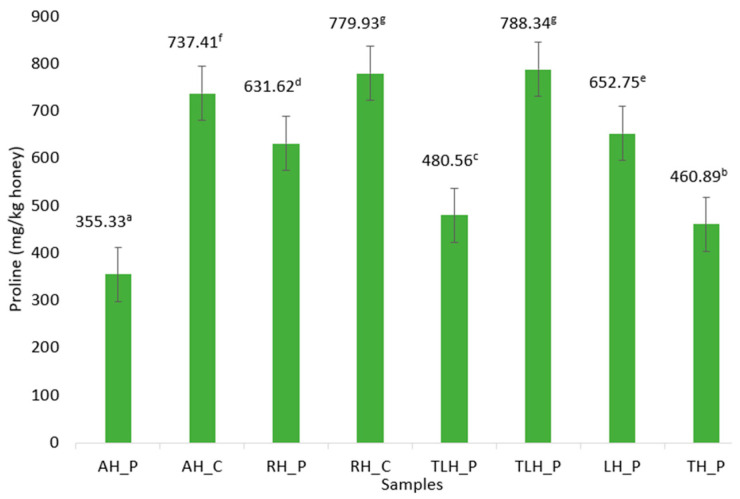
The proline content (mg/kg) of the honey samples. The results are presented as the means ± SD (n = 3). ^a–g^—the different lowercase letters indicate significant differences between the samples (*p* < 0.05) according to Turkey’s multiple comparison test. Samples sharing the same letter are not significantly different from each other. AH_P, producer acacia honey; AH_C, commercial acacia honey; RH_P, producer rapeseed honey; RH_C, commercial rapeseed honey; TLH_P, producer linden honey; TLH_P, commercial linden honey; LH_P, producer lavender honey; TH_P, producer thyme honey.

The highest levels of proline were observed in the RH_C (779.93 mg/kg) and TLH_C (788.34 mg/kg) samples, closely followed by AH_C (737.41 mg/kg). LH_P (652.75 mg/kg) and RH_P (631.62 mg/kg) also exhibited high proline levels, suggesting a floral origin naturally rich in amino acids. In contrast, TH_P (460.89 mg/kg) and TLH_P (480.56 mg/kg) had significantly lower levels, though still above the quality threshold of 180 mg/kg. The lowest proline content was recorded in the producer-sourced AH_P (355.33 mg/kg). All the measured values were well above the minimum quality threshold (180 mg/kg). As indicated by the distinct lowercase letters in Figure 6, statistically significant differences (*p* < 0.05) were observed not only between floral varieties, but also between samples from different sourcing backgrounds (producer vs. commercial).

### 3.7. Sensory Evaluation

**Figure 7:**
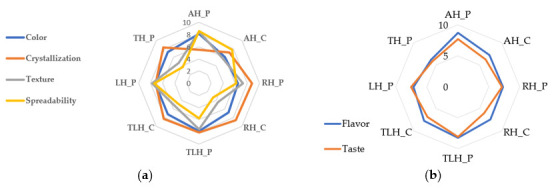
Sensory perception of monofloral honey samples by untrained panelists: (**a**) visual and tactile characteristics; (**b**) gustatory and olfactory characteristics. AH_P, producer acacia honey; AH_C, commercial acacia honey; RH_P, producer rapeseed honey; RH_C, commercial rapeseed honey; TLH_P, producer linden honey; TLH_P, commercial linden honey; LH_P, producer lavender honey; TH_P, producer thyme honey.

The sensory evaluation focused on two main categories: visual and tactile attributes (color, crystallization, texture, and spreadability) and gustatory–olfactory characteristics (flavor and taste). As shown in Figure 7a, the AH_P sample consistently received the highest ratings across all physical attributes: color (7.97 ± 0.85), texture (8.37 ± 0.81), and spreadability (8.43 ± 0.63). Its commercial counterpart, AH_C, also performed well, but showed slightly lower scores, particularly in texture (6.03 ± 0.96) and color (5.90 ± 0.96), suggesting a denser consistency and a less vibrant appearance. The rapeseed (RH_C, RH_P) and linden (LH_C, LH_P) honeys had lower ratings for spreadability and crystallization. For example, RH_C’s spreadability was rated at just 3.50 ± 0.86, reflecting a firmer, more crystallized texture that may not appeal to all consumers. The thyme (TH_P) and lavender (TLH_P) honeys showed balanced profiles, with moderate scores across the board, making them solid but less standout options. Overall, local products consistently outperformed commercial ones in physical traits, supporting the idea that provenance matters when it comes to perceived honey quality.

Turning to the flavor and taste, the results (Figure 7b) revealed even more pronounced differences. Once again, AH_P led the group with the highest scores: flavor at 8.57 ± 0.68 and taste at 7.60 ± 1.00. Its commercial version, AH_C, followed closely but fell short, especially in taste (6.40 ± 1.00), reinforcing consumer preference for fresh, local honey. By contrast, rapeseed and linden honeys, both producer and commercial, were consistently rated lower. RH_C, for instance, received just 5.97 ± 1.30 for taste, pointing to a milder or less appealing profile. Lavender and thyme honeys performed better on aroma than taste; TLH_P, for example, scored 8.13 ± 0.82 for flavor but only 5.93 ± 1.08 for taste (Table 5).

### 3.8. Correlation Analysis

**Figure 8:**
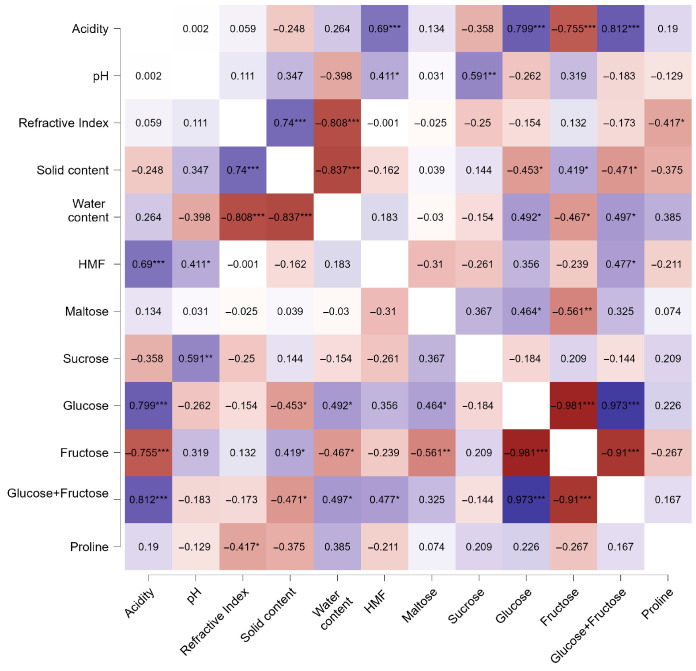
Correlation matrix of physicochemical and sugar parameters in honey samples.Significance levels: * *p* < 0.05; ** *p* < 0.01; *** *p* < 0.001.

The relationships among the physicochemical parameters of the honey samples were examined using Pearson correlation analysis. The resulting heatmap (Figure 8) illustrates the strength and direction of the linear associations between variables such as acidity, pH, refractive index, water and solid content, HMF, individual sugars (glucose, fructose, sucrose, maltose), glucose + fructose, and proline. A very strong negative correlation was observed between glucose and fructose (r = −0.984, *p* < 0.001), which reflects their inverse balance in honey composition and their role in crystallization behavior. Fructose also showed a very strong negative correlation with glucose + fructose content (r = −0.91, *p* < 0.001), consistent with its role as a primary monosaccharide in honey.

Acidity was positively correlated with both glucose (r = 0.798, *p* < 0.05) and glucose + fructose (r = 0.812, *p* < 0.05), suggesting that higher sugar content may enhance acid formation through metabolic or enzymatic pathways. The refractive index correlated strongly and positively with solid content (r = 0.842, *p* < 0.01) and negatively with water content (r = −0.842, *p* < 0.001), indicating a direct physical relationship between honey’s moisture and density. Although water content was negatively associated with glucose (r = −0.506) and glucose + fructose (r = −0.457), these correlations were not statistically significant (*p* > 0.05). No significant associations were found between proline and any of the other measured parameters, suggesting that its variability was not directly linked to basic physicochemical traits (Figure 8).

**Figure 9:**
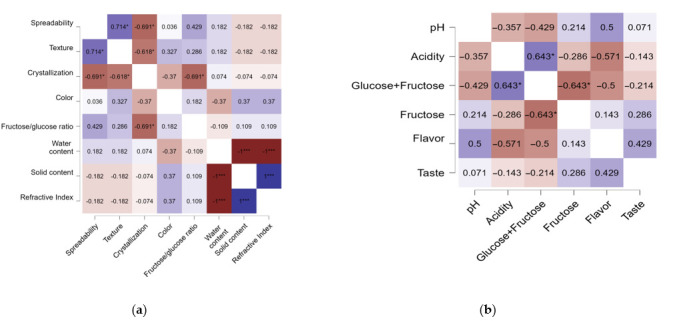
Kendall’s Tau correlations between (**a**) visual and tactile characteristics and physicochemical properties of honey samples; (**b**) flavor and taste characteristics and physicochemical properties of honey samples.Significance levels: * *p* < 0.05; *** *p* < 0.001.

To explore the link between sensory characteristics and physicochemical properties, Kendall’s Tau correlation was used. As shown in Figure 9a, significant associations were observed between visual–tactile attributes and analytical parameters such as refractive index, moisture and solid content, and fructose-to-glucose (F/G) ratio.

Crystallization showed a strong negative correlation with both ease of spreading (τ = −0.691, *p* < 0.05) and texture (τ = −0.618, *p* < 0.05), indicating that more crystallized honey is perceived as less smooth and more difficult to spread. The F/G ratio was positively correlated with spreadability (τ = 0.429) and negatively correlated with crystallization (τ = −0.691, *p* < 0.05), supporting the known effect of higher fructose contents in reducing crystallization and enhancing fluidity. No significant correlations were identified between color and any of the measured physicochemical traits. As expected, perfect correlations (τ = ±1) were observed among water content, solids, and refractive index, due to their mathematically interdependent nature.

Kendall’s Tau analysis between chemical parameters (pH, acidity, glucose + fructose content) and taste–olfactory perceptions is shown in Figure 9b. A moderate positive correlation was found between taste and flavor (τ = 0.429), indicating that honeys with stronger aromas were generally rated as more flavorful. A weaker, yet positive, correlation was also observed between taste and fructose content (τ = 0.286), supporting the role of fructose in enhancing sweetness and palatability, as it is known to be sweeter than glucose.

Furthermore, aroma correlated positively with pH (τ = 0.429) and negatively with acidity (τ = −0.571), suggesting that samples with lower acidity and higher pH values were more favorably perceived in terms of aroma. These findings underscore the importance of acid–base balance in shaping the sensory profile of honey. A negative correlation between glucose + fructose and fructose (τ = −0.643) was also observed, potentially reflecting variability in sugar composition across floral types.

## 4. Discussion

In light of increasing concerns regarding honey authenticity and the variability introduced by floral and geographic origin, this study offers a multidimensional perspective on honey quality through the integration of physicochemical and sensory data. The inclusion of both producer and commercial samples was intended to allow for a direct comparison between minimally handled honey and products that may have undergone filtration, pasteurization, or prolonged storage. This strategy also reflects the dual pathways through which consumers typically access honey in Romania, direct purchases from small-scale beekeepers versus pre-packaged honey available in stores.

Pollen morphology, characterized by grain symmetry, aperture type, exine ornamentation, and staining behavior, enabled classification the of the eight monofloral honey samples into five well-defined botanical types. The aperture patterns identified in our honey samples align well with previously reported morphological variations. The findings are in agreement with published palynological data and conform to criteria established in the European Pharmacopoeia (10th Edition) [36]. In particular, the identification of hexacolporate pollen grains in thyme honey corresponds with established records for certain *Thymus* taxa. These aperture characteristics, though less common within *Lamiaceae,* are documented in specialized palynological resources, such as the PalDat database [37], and support the morphological classification observed in our analysis.

Acacia honey samples contained exclusively *Robinia pseudoacacia* pollen, with no secondary species detected, supporting a highly pure monofloral status. This is typical for acacia honeys produced in regions dominated by black locust trees and minimal floral competition, a pattern also confirmed by other profiling studies on Romanian and Central European honeys [38,39]. Similarly, rapeseed honeys showed the uniform presence of *Brassica napus* pollen in both producer and commercial samples. The high pollen productivity of this species and its seasonal dominance often result in monofloral honeys, as noted in multiple sensory and melisso-palynological studies across Europe [39,40]. In contrast, the linden, lavender, and thyme honeys contained small proportions of secondary pollen types such as *Helianthus annuus*, *Cichorium intybus*, or *Salvia species*. These are likely attributable to overlapping flowering periods and shared foraging territories. Nevertheless, the dominance of *Tilia*, *Lavandula angustifolia*, and *Thymus* pollen in the respective samples confirms their monofloral character. These observations are consistent with findings from studies on Iberian honeys, which concluded that minor secondary pollen presence does not compromise monofloral classification when the principal floral source remains dominant [41].

No significant morphological differences were observed between samples from producers and those from commercial sources within the same floral groups, suggesting a degree of consistency in floral sourcing for the samples analyzed. However, these observations are limited to the specific honeys and regions included in this study. Given the relatively small and geographically constrained sample size, broader conclusions regarding supply chain integrity or processing impact cannot be robustly supported. Moreover, the absence of quantitative pollen analysis further restricts the ability to confirm floral origin according to standardized thresholds. According to EU Directive 2014/63/EU [42], monofloral designation typically requires that the dominant pollen type exceeds 45% of the total pollen content. Although our morphological identification revealed either the exclusive or clearly predominant presence of the declared floral pollen in all samples, we acknowledge that this qualitative approach cannot substitute for formal quantification. Future studies should combine both qualitative and quantitative palynological analyses to achieve full regulatory compliance and increase botanical traceability.

When combined with compositional and sensory assessments, pollen analysis enhances the traceability, authenticity, and market value of honey. It also contributes to protecting consumer trust and supporting producers who rely on floral integrity as a mark of product identity [19,43].

Free acidity, determined by the presence of organic acids, lactones, phenolic compounds, and inorganic ions, is one of the most relevant indicators of honey freshness and quality. Elevated acidity may suggest fermentation or degradation, while values below the EU regulatory threshold of 50 mEq/kg confirm product stability [5,6,44]. In our study, acidity levels varied significantly across floral types (*p* < 0.001), with acacia honeys consistently exhibiting the lowest acidity and lowest pH, aligning with their well-known mildness and reduced organic acid content. By contrast, the rapeseed, lavender, and thyme honeys showed substantially higher acidity levels, indicating a more active biochemical composition. Linden honey stood out with a moderate acidity profile but the highest pH value. These findings are consistent with previous studies that highlighted strong floral influence on honey acidity and pH [19,45,46].

Moisture and refractive index values also contributed valuable insights. All samples complied with the Codex Alimentarius threshold of 20% moisture content, confirming their adequate preservation [5,6]. However, variability was observed, with thyme honey presenting the lowest moisture and highest refractive index, indicating full maturation and minimal fermentation risk. In contrast, the producer samples of rapeseed and lavender displayed slightly elevated moisture, suggesting premature extraction or insufficient dehydration, an issue also noted in earlier studies [47,48].

The HMF content, a recognized marker of thermal exposure and storage degradation, remained well below the EU maximum limit of 40 mg/kg [49,50] across all samples. Notably, some producer samples showed significantly higher HMF levels (*p* < 0.05) than their commercial counterparts. Although no detailed records of processing or storage conditions were available, this finding may reflect factors such as extraction at elevated temperatures, longer ambient storage before testing, or limited standardization in handling at the small-scale producer level. The acacia honeys consistently recorded the lowest HMF concentrations, affirming their chemical stability and minimal processing susceptibility. The highest HMF level was observed in a linden sample from a producer, though still within legal limits, possibly reflecting seasonal variation or non-optimal storage. These results are aligned with other regional and international findings showing variability in HMF depending on floral source and handling [19,51,52].

Carbohydrate analysis further elucidated floral influence on honey composition. As expected, fructose was the dominant sugar, followed by glucose. All samples complied with regulatory standards, showing sucrose contents below the maximum threshold of 5 g/100 g, as stipulated by Codex Alimentarius and EU regulations [53,54]. The F/G ratio, a predictor of crystallization behavior, varied significantly among honey types. Acacia honey, with the highest F/G ratio, is known for its low crystallization rate and enhanced sweetness, properties confirmed in this study. Rapeseed honey had the lowest F/G ratio and a high glucose content (>42 g/100 g), explaining its tendency to crystallize rapidly, a trend mirrored in both Romanian and Indian honey studies [55].

Lavender and thyme honeys displayed intermediate F/G ratios, suggesting moderate granulation potential and balanced texture, consistent with Mediterranean monofloral profiles [56]. The slightly higher sucrose and maltose concentrations in some commercial linden and rapeseed honeys may reflect incomplete enzymatic inversion or earlier harvesting, a hypothesis supported by other studies observing similar patterns in market samples [57]. Glucose + fructose content also varied, with rapeseed honey from producer sources showing the highest values, likely due to its denser sugar matrix. Conversely, acacia honeys had slightly lower combined glucose and fructose levels, in line with their lighter sensory and compositional profiles.

Taken together, these findings underscore the critical role of floral origin in shaping honey’s acidity, sugar profile, and fermentation potential. Moreover, parameters such as HMF and moisture not only reflect freshness but also indicate the impact of processing and storage practices. The integration of these indicators supports a robust framework for honey quality evaluation, authenticity assurance, and regulatory compliance.

The glycemic index is an important nutritional parameter, particularly relevant for individuals with impaired glucose tolerance or diabetes. In this study, the eGI of the eight monofloral honey samples was evaluated using a theoretical model based on the glycemic behavior of individual sugars (glucose, fructose, sucrose, and maltose). The analysis revealed a wide range of eGI values, from moderate to high, influenced by the specific sugar composition of each sample. One of the most relevant findings was the strong inverse relationship between the fructose-to-glucose (F/G) ratio and eGI. Honeys with higher fructose contents consistently demonstrated lower estimated glycemic impact. This observation aligns with established metabolic pathways: fructose is absorbed more slowly and primarily metabolized in the liver, resulting in a reduced glycemic response compared to glucose [28,29].

Statistical validation of this relationship using Pearson correlation confirmed a very strong negative association between F/G ratio and eGI (r = −0.98, *p* < 0.001). This reinforces the visual trends observed in the scatterplot and underscores the predictive value of the F/G ratio as a proxy for glycemic behavior. These results are consistent with both theoretical models and previous empirical findings that link higher fructose content with attenuated postprandial glycemia [30,31]. From a nutritional perspective, these findings suggest that honey should not be regarded as a uniform glycemic agent. Its glycemic response is highly dependent on monosaccharide composition, which, in turn, is shaped by botanical origin [58]. Overall, the results highlight the need to consider botanical origin and sugar composition when assessing the metabolic suitability of honey. Not all honeys are metabolically equivalent, and their impact on glycemia may differ significantly depending on intrinsic composition. Future research should aim to validate these findings through clinical studies and explore the potential synergistic effects of honey’s bioactive compounds, such as polyphenols and organic acids, on glycemic modulation.

Beyond sugar composition, nitrogenous compounds such as proline provide valuable biochemical markers for honey maturity, floral origin, and processing integrity. In the present study, all the honey samples displayed proline concentrations above the commonly accepted minimum threshold of 180 mg/kg, suggesting adequate ripening. The commercial linden and rapeseed honeys exhibited significantly higher proline levels (*p* < 0.01), which may reflect differences in maturation stage, hive management practices, or handling conditions along the supply chain [59,60]. Conversely, acacia honey from producers showed the lowest proline concentrations, an observation aligned with the naturally low proline yields of *Robinia pseudoacacia* honeys. Prior studies have reported similar trends, with acacia samples consistently presenting lower proline levels compared to other monofloral types such as jujube or multifloral honeys [59]. These variations highlight the combined influence of botanical origin, bee foraging behavior, and post-harvest handling on the nitrogenous composition of honey. The elevated proline levels observed in some commercial samples may result from multiple factors, including advanced maturation or longer time frames between harvesting and packaging. While these patterns support the role of proline as a valuable marker of honey quality and authenticity, further research with controlled processing variables is needed to clarify the underlying mechanisms.

Sensory characteristics—aroma, flavor, texture, and appearance—are critical to consumer preference and honey’s market value. In this study, perceptual differences were evident across floral types and sources. AH_P received the highest scores in terms of color, aroma, taste, spreadability, and texture, while the commercial linden and rapeseed honeys were rated lower, primarily due to their crystallization and diminished flavor clarity. These findings echo previous studies showing that floral origin and minimal processing significantly enhance sensory quality [59,61]. For instance, the thyme and lavender honeys were perceived as more aromatic than flavorful, indicating that aroma intensity may be preserved better than taste during storage.

To explore the interdependence of chemical and sensory traits, a comprehensive correlation analysis was conducted. Glucose + fructose content correlated positively with acidity (*p* < 0.05), a finding previously reported in studies linking sugar concentration to organic acid accumulation [62]. A strong inverse relationship between glucose and fructose (*p* < 0.001) was also observed, reflecting the monosaccharide balance that governs crystallization behavior, a phenomenon well documented in regional honey types [45]. Water content correlated negatively with solid content and refractive index (*p* < 0.001), confirming established models used to assess honey concentration and shelf stability [63]. Proline content did not correlate significantly with other physicochemical parameters, highlighting its dependence on floral origin and bee metabolism, as also noted in Algerian honey studies [64].

Kendall’s Tau correlation further revealed important associations between sensory and chemical properties. Spreadability and texture showed a strong positive correlation, while both were negatively associated with crystallization, confirming that high-glucose honeys tend to be firmer and less spreadable [65]. The F/G ratio demonstrated a strong inverse relationship with crystallization tendency, in line with previous observations of tropical and Mediterranean honey types [66,67]. Honeys with higher glucose levels consistently exhibited more rapid granulation and firmer textures. Moreover, acidity correlated negatively with flavor and taste, suggesting that highly acidic honeys are perceived as less pleasant. Fructose content was negatively correlated with glucose + fructose and taste, indicating a potential dilution effect on perceived sweetness when fructose dominates the sugar profile, an effect previously documented in Brazilian monofloral honeys [68].

These integrated findings confirm that honey’s sensorial and physicochemical traits are deeply interlinked. Botanical origin remains the dominant influence across all quality dimensions, from sugar composition to flavor perception.

The integrated evaluation of proline, HMF content, acidity, sugar composition, and sensory attributes enabled a detailed and multidimensional characterization of the analyzed monofloral honey samples. By including both producer-sourced and commercially available products, this study reflects real-world diversity and reveals potential differences linked to harvesting practices, processing intensity, and storage conditions across the supply chain. The application of correlation and multivariate statistical analyses significantly strengthened the interpretation of the results, demonstrating the discriminatory capacity of key quality markers in distinguishing honeys based on their floral origin. While the scope of the study was limited to a specific number of floral types and regions within Romania, the findings remain relevant for broader efforts aimed at honey standardization and market transparency.

However, this study has several limitations that should be acknowledged. First, the analysis focused on a selected number of floral types and regions within Romania, which may narrow the generalizability of the findings. Commercial samples of lavender and thyme honeys could not be included, as these products were not available on the Romanian retail market at the time of sampling. Consequently, comparisons between producer and commercial sources were limited to the other floral types. The pollen analysis was conducted qualitatively, without applying quantitative thresholds for dominant pollen, although the exclusive or clearly predominant presence of floral-specific pollen types offered reliable support for monofloral classification. Regarding sensory evaluation, the panel consisted of young adult consumers with no formal training; while participants received guidance prior to tasting, the results reflect consumer perceptions rather than expert sensory profiling. Additionally, the study did not include compositional markers such as enzyme activities, protein degradation, or volatile compounds, which could have provided more direct insights into the impact of processing and storage. Although statistical comparisons were made for HMF and proline levels, these parameters alone are not sufficient to determine processing intensity. Information on pre-sale handling and storage conditions, especially for the commercial samples, was unavailable and may have influenced some of the observed values. Nevertheless, the results contribute to the growing body of evidence supporting the role of integrated physicochemical and sensory profiling in verifying the botanical origin, supporting the traceability, and promoting the valorization of natural, minimally processed honey.

## 5. Conclusions

The results highlight the influence of botanical origin on honey’s chemical composition, sensory attributes, and estimated glycemic behavior. Parameters such as proline content, HMF levels, acidity, and fructose/glucose ratio proved particularly useful in differentiating floral types and assessing product quality. Producer-sourced honeys showed more favorable sensory profiles, while market samples exhibited signs of higher processing intensity. The strong correlations identified between chemical and sensory parameters emphasize the importance of using combined analytical approaches for authenticity verification and product evaluation. These findings support the use of floral-origin-specific quality markers to enhance honey traceability, support consumer trust, and promote the valorization of authentic, minimally processed honey in both local and commercial contexts.

## Figures and Tables

**Table 1:** Honey samples classified by botanical origin, source type, and collection region.

Sample Code	Botanical Origin	Source Type	Collection Region
AH_P	Acacia (*Robinia pseudoacacia* L.)	Producer	Brăila County
AH_C		Commercial	National retail
RH_P	Rapeseed (*Brassica napus* L.)	Producer	Buzău County
RH_C		Commercial	National retail
TLH_P	Linden (*Tilia* spp.)	Producer	Buzău County
TLH_C		Commercial	National retail
LH_P	Lavender (*Lavandula angustifolia* L.)	Producer	Vaslui County
TH_P	Thyme (*Thymus* spp.)	Producer	Sălaj County

P: Producer sample, raw and unprocessed, harvested in 2022; C: commercial sample purchased from Romanian retail market.

**Table 4:** The carbohydrate content (g/100 g) of the honey samples.

Pk	Rt (min)	Compound	AH_P	AH_C	RH_P	RH_C	TLH_P	TLH_C	LH_P	TH_P
1	8.37	Maltose	1.423 ± 0.01 ^e^	0.496 ± 0.01 ^a^	1.445 ± 0.01 ^e^	1.595 ± 0.02 ^f^	0.929 ± 0.01 ^c^	1.663 ± 0.01 ^g^	0.616 ± 0.01 ^b^	1.038 ± 0.01 ^d^
2	9.16	Sucrose	2.822 ± 0.01 ^c^	3.103 ± 0.01 ^e^	3.024 ± 0.01 ^d^	3.217 ± 0.02 ^f^	3.404 ± 0.01 ^g^	3.107 ± 0.01 ^e^	1.983 ± 0.01 ^a^	2.121 ± 0.01 ^b^
3	10.55	Glucose	22.841 ± 0.1 ^b^	20.277 ± 0.01 ^a^	42.354 ± 0.2 ^h^	31.520 ± 0.1 ^e^	25.426 ± 0.1 ^c^	31.988 ± 0.2 ^f^	30.494 ± 0.1 ^d^	33.626 ± 0.1 ^g^
4	11.27	Fructose	47.151 ± 0.1 ^e^	49.507 ± 0.2 ^f^	37.746 ± 0.1 ^a^	42.696 ± 0.2 ^c^	47.305 ± 0.1 ^e^	41.589 ± 0.2 ^b^	43.592 ± 0.2 ^d^	41.736 ± 0.2 ^b^
Fructose + glucose			69.99 ± 0.14 ^b^	69.78 ± 0.14 ^a^	80.10 ± 0.22 ^e^	74.22 ± 0.22 ^d^	72.73 ± 0.14 ^c^	73.58 ± 0.22 ^d^	74.09 ± 0.22 ^c^	75.36 ± 0.22 ^d^
Fructose/glucose ratio			2.06	2.44	0.89	1.35	1.86	1.3	1.43	1.24

Pk, peak; Rt-retention time; AH_P, producer acacia honey; AH_C, commercial acacia honey; RH_P, producer rapeseed honey; RH_C, commercial rapeseed honey; TLH_P, producer linden honey; TLH_P, commercial linden honey; LH_P, producer lavender honey; TH_P, producer thyme honey. ^a–f^—different lowercase letters indicate significant differences between samples (*p* < 0.05) according to Turkey’s multiple comparison test. Samples sharing same letter are not significantly different from each other.

**Table 5:** Mean hedonic scores (±SD) of sensory attributes.

Sample	Color	Crystallization	Spreadability	Texture	Flavor	Taste
AH_C	5.9 ± 0.96 ^a^	7.3 ± 1.02 ^b^	7.77 ± 0.82 ^e,f^	6.03 ± 0.96 ^c^	7.47 ± 0.82 ^b,c^	6.4 ± 1.0 ^a,b^
AH_P	7.97 ± 0.85 ^e^	5.3 ± 1.06 ^a^	8.43 ± 0.63 ^f^	8.37 ± 0.81 ^e^	8.57 ± 0.68 ^d^	7.6 ± 1.0 ^c,d^
LH_P	7.2 ± 0.92 ^c,d^	7.03 ± 1.13 ^b^	7.63 ± 0.93 ^e^	7.63 ± 0.93 ^d,e^	7.2 ± 1.03 ^b^	7.43 ± 0.94 ^c,d^
RH_C	6.87 ± 0.97 ^b,c^	8.57 ± 0.73 ^c^	3.5 ± 0.86 ^a^	4.23 ± 0.97 ^a^	7.67 ± 0.88 ^b,c^	5.97 ± 1.3 ^a^
RH_P	6.43 ± 1.04 ^a,b^	8.7 ± 0.53 ^c^	6.57 ± 0.97 ^d^	7.3 ± 1.26 ^d^	7.1 ± 0.8 ^b^	7.4 ± 1.1 ^c,d^
TH_P	7.3 ± 0.95 ^c,d,e^	8.17 ± 0.83 ^c^	4.0 ± 1.05 ^a^	4.97 ± 0.85 ^a,b^	5.93 ± 1.08 ^a^	5.93 ± 1.11 ^a^
TLH_C	7.57 ± 0.94 ^c,d,e^	8.17 ± 0.83 ^c^	4.8 ± 1.16 ^b^	5.4 ± 1.16 ^b,c^	7.6 ± 1.07 ^b,c^	7.0 ± 1.02 ^b,c^
TLH_P	7.7 ± 0.95 ^d,e^	8.27 ± 0.83 ^c^	5.77 ± 1.01 ^c^	7.6 ± 0.89 ^d,e^	8.13 ± 0.82 ^c,d^	7.97 ± 1.03 ^d^

AH_P, producer acacia honey; AH_C, commercial acacia honey; RH_P, producer rapeseed honey; RH_C, commercial rapeseed honey; TLH_P, producer linden honey; TLH_P, commercial linden honey; LH_P, producer lavender honey; TH_P, producer thyme honey, ^a–f^—different lowercase letters indicate significant differences between samples (*p* < 0.05) according to Turkey’s multiple comparison test. Samples sharing the same letter are not significantly different from each other.

## Data Availability

The original contributions presented in the study are included in the article, further inquiries can be directed to the corresponding author.

## Abbreviations

The following abbreviations are used in this manuscript:

AH	Acacia honey (*Robinia pseudoacacia* L.)
AH_C	Acacia honey commercial
AH_P	Acacia honey producer
C	Commercial
eGI	Estimated glycemic index
EU	European Union
F	Fructose
G	Glucose
GI	Glycemic index
HMF	Hydroxymethyl furfural
HPLC-RID	High-Performance Liquid Chromatography with Refractive Index Detection
H_2_SO_4_	Sulfuric acid
IHC	International Honey Commission
LH	Lavender honey (*Lavandula angustifolia* L.)
LH_P	Producer lavender honey
M	Maltose
mEq	Milliequivalent
mg	Milligram
mL	Milliliter
P	Producer
PCA	Principal Component Analysis
RH	Rapeseed honey (*Brassica napus* L.)
RH_C	Commercial rapeseed honey
RH_P	Producer rapeseed honey
S	Sucrose
SD	Standard deviation
TA	Titratable acidity
TH	Breckland Thyme honey (*Thymus serpyllum* L.)
TH_C	Commercial Breckland Thyme honey
TH_P	Producer Breckland Thyme honey
TLH	Linden honey (*Tilia* spp.)
TLH_C	Commercial linden honey
TLH_P	Producer linden honey
TS	Total solids
USD	United States dollar
UV-vis	Ultraviolet–visible
